# The presence of *Plasmodium malariae* and *Plasmodium knowlesi* in near malaria elimination setting in western Indonesia

**DOI:** 10.1186/s12936-022-04335-y

**Published:** 2022-11-05

**Authors:** Irbah Rea Alvieda Nainggolan, Rycha Dwi Syafutri, Monica Nadya Sinambela, Clara Devina, Handayani, Beby Syofiani Hasibuan, Sriwipa Chuangchaiya, Paul C. S. Divis, Zulkarnain Md Idris, Ranti Permatasari, Inke Nadia Diniyanti Lubis

**Affiliations:** 1 Faculty of Medicine, Universitas Sumatera Utara, Medan, 20155 Indonesia; 2 Department of Paediatrics, Faculty of Medicine, Universitas Sumatera Utara, Medan, 20155 Indonesia; 3 Department of Community Health, Faculty of Public Health, Kasetsart University, Chalermphrakiat Sakon Nakhon Province Campus, 47000 Sakon Nakhon, Thailand; 4 Malaria Research Centre, Faculty of Medicine and Health Sciences, Universiti Malaysia Sarawak, 94300 Kota Samarahan, Sarawak Malaysia; 5 Department of Parasitology and Medical Entomology, Faculty of Medicine, Universiti Kebangsaan Malaysia, 56000 Cheras, Kuala Lumpur, Malaysia; 6 Department of Clinical Pathology, Faculty of Medicine, Universitas Sumatera Utara, Medan, 20155 Indonesia

**Keywords:** Malaria, Indonesia, North Sumatera, Elimination, *P. knowlesi*, *P. malariae*

## Abstract

**Background:**

Indonesia is progressing towards malaria elimination. To achieve this goal, intervention measures must be addressed to cover all *Plasmodium* species. Comprehensive control measures and surveillance programmes must be intensified. This study aims to determine the prevalence of microscopic and submicroscopic malaria in Langkat district, North Sumatera Province, Indonesia.

**Methods:**

A cross-sectional survey was conducted in six villages in Langkat district, North Sumatera Province in June 2019. Data were recorded using a standardized questionnaire. Finger pricked blood samples were obtained for malaria examination using rapid diagnostic test, thick and thin blood smears, and polymerase chain reaction.

**Results:**

A total of 342 individuals were included in the study. Of them, one (0.3%) had a microscopic *Plasmodium malariae* infection, no positive RDT examination, and three (0.9%) were positive for *P. malariae* (n = 1) and *Plasmodium knowlesi* (n = 2). The distribution of bed net ownership was owned by 40% of the study participants. The participants had a house within a radius of 100–500 m from the forest (86.3%) and had the housing material of cement floor (56.1%), a tin roof (82.2%), wooden wall (35.7%), bamboo wall (28.1%), and brick wall (21.6%).

**Conclusion:**

Malaria incidence has substantially decreased in Langkat, North Sumatera, Indonesia. However, submicroscopic infection remains in the population and may contribute to further transmission. Surveillance should include the detection of microscopic undetected parasites, to enable the achievement of malaria elimination.

## Background

Indonesia has shown significant progress towards a malaria-free zone within the last decade with a reduction in 50% of cases and 66% of deaths due to malaria [1]. Aggressive efforts in scaling up diagnostic and treatment measures with a tailored intervention for each district of the country are essential to achieve malaria-free by 2030 [2, 3]. Nevertheless, despite the effort to scale up control measures by the government, there were still an estimated 784,854 cases and 1443 deaths due to malaria in 2020 [4]. Furthermore, in 2020, Indonesia is one of two countries in Southeast Asia failing to achieve the WHO Global Technical Strategy with 40% reduction of cases and deaths compared to 2015 [5].

Microscopic examination is currently the gold standard for malaria diagnosis, however, misdiagnosis occurs in cases of low parasitaemia as well as incorrect species identification [6]. Rapid diagnostic test (RDT) is commonly used to diagnose malaria in remote areas where expertise are not available [7], and for lower density infections, polymerase chain reaction (PCR) has higher sensitivity and is used extensively for malaria surveillance and research, yet it has limitations as a routine diagnostic method [8].

In near to elimination settings, there are several challenges to approach its elimination goal. There is a shift in the trend of malaria infection with a decrease of *Plasmodium falciparum* infection prevalence to an increase of non-*P. falciparum* infection prevalence. This is a concern as the current malaria elimination strategies are focusing on *P. falciparum* and *Plasmodium vivax* infections [9], and the current control measures might not be suitable to reduce other non-falciparum and non-vivax infections. Furthermore, the submicroscopic infection is a major malaria elimination challenge that is manifested without any symptoms but remains as a source of transmission for malaria infection [10]. In low transmission areas, microscopic examination misses a considerable proportion of parasites due to submicroscopic infections that persist longer because of the absence of proper treatment [10, 11].

The prevalence of submicroscopic malaria infection in Langkat was 33.5% with falciparum malaria infection being the most common type of malaria infection in that area [12]. This study aims to describe the prevalence of malaria infection both by microscopic and submicroscopic examination. The results of this study can inform the stakeholders of the importance of molecular surveillance in the regions where malaria is almost eliminated.

## Methods

### Ethics statement

The study was conducted in accordance with the Declaration of Helsinki and was approved by the Ethics Committee of the Faculty of Medicine, Universitas Sumatera Utara (No. 179/TGL/KEPK FK-USU-RSUPHAM/2019). Survey subjects were informed by the local health district personnel of the purpose and procedures of the study, and a written informed consent was obtained from each adult participant at study registration. In the case of individuals < 18 years, written informed consents were sought from their parents or legal guardians.

### Study sites and sample collection

**Fig. 1:**
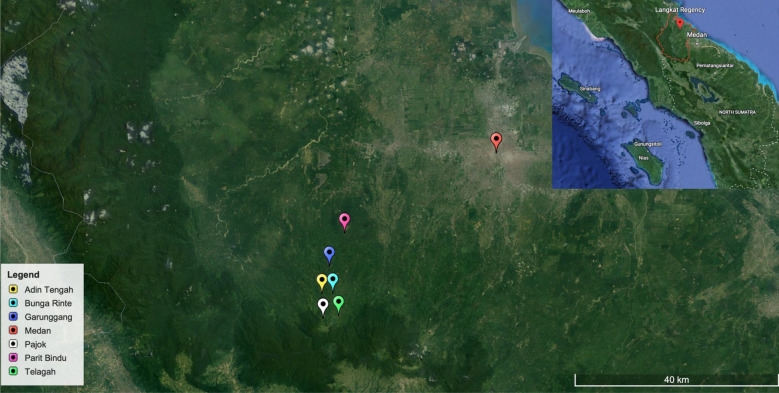
Map of study setting in Langkat district, North Sumatera Province, Indonesia where malaria surveillance was conducted

A cross-sectional study was conducted in six villages (i.e. Garunggang, Telagah, Bunga Rinte, Parit Bindu, Adin Tengah and Pajok) in Langkat district, North Sumatera Province, Indonesia in June 2019 (Fig. 1). Langkat has an area of 6263.29 km^2^ with an altitude of 4–105 m above sea level. The population of Langkat was estimated 1,041,775 inhabitants in 2019 [13]. All villages have the same environmental characteristics with their location on the forest fringes. Farming is the major economic activity for the villagers with major crops including rice and vegetables.

The sample size for study participants was calculated using the following Cochran’s formula: N = z^2^ p (1 − p)/e2, where z is the confidence interval which is set at 95% (z-value of 1.96); (p) is the expected prevalence of malaria infections of 34% from a previous study [12] and (e) is the allowed error margin which is set to 5%. In addition, contingencies were adjusted by adding another 10% of individuals, giving a minimum of 334 participants to be sampled. A convenience sampling strategy was used in this study. The village leaders and household heads were informed about the objectives and procedures of the study, and were asked to invite residents and family members to come to the selected survey point to participate. The inclusion criteria were individuals aged over 6 months old, had been living in the area for at least 6 months and consented to participate in the study. Meanwhile, the exclusion criteria were physically or mentally unfit individuals and incomplete examination data. A standardized questionnaire was used to collect sociodemographic information from each participant.

### Field and laboratory methods

Blood sample was collected from finger pricked and spotted on glass slides for thick and thin blood film, onto malaria RDT (CareStart™ Malaria HRP2/pLDH [Pf/PAN], AccessBio Inc, USA), onto the Whatman 3MM filter paper (Whatman International, UK) for molecular screening, and onto a microcuvette for Hb examination using the HemoCue® Hb 301 (HemoCue, Sweden). The malaria RDT kit and Hb examination were performed according to the manufacturer’s instructions and read on site. Similarly, thin and thick blood films were prepared on site, stored in slide boxes and transported to the Parasitology Laboratory, at the Faculty of Medicine, Universitas Sumatera Utara. Thin blood films were fixed with methanol and all films were stained with 3% Giemsa solution (Sigma-Aldrich, Germany) for 30 min and examined under oil immersion (10× 100× magnification) by experienced microscopists. Blood films were defined as negative if no parasites were found after examining 1000 × high power microscopy fields. For any positive samples, malaria species were identified and asexual parasite forms were counted against 200 leukocytes. Gametocytes counts were separately recorded. Parasite density was estimated from parasite counts, assuming that there were 8000 leukocytes per μl of blood. All slides were independently re-examined by two experienced microscopists blinded to the first microscopy reading results. Discrepancies between the two readings were resolved by a third experienced microscopist.

For the detection by molecular screening, blood spotted filter papers were air-dried thoroughly at ambient temperature before sealed in individual zipped plastic bags and stored at the − 20 °C. The molecular analysis was performed at the laboratory in the Faculty of Medicine, Universitas Sumatera Utara, Medan. DNA was extracted using Chelex method [14], and PCR amplification for *Plasmodium* spp. identification was conducted following previously published methods targeting the 18S rRNA, and confirmation of *P. knowlesi* infection was done using PCR targeting the *sicavar* genes [12, 15, 16].

### Statistical analysis

The data collected were double entered into Microsoft Excel (version 2016) spreadsheets and cross-checked for errors. Then, the data were processed and analysed using SPSS software (IBM, version 25). The data were presented as a frequency distribution table and analysed with Fisher's exact test (P ≤ 0.05) to identify the risk factors of submicroscopic malaria as well the correlation with related variables evaluated.

## Results

### Characteristic of respondents

A total of 342 participants were enrolled in this study (Table 1). The majority of participants were female (68.4%), aged > 18 years (75.4%) with a median age 39 years old, and belong to Batak Karo ethnicity (97.7%). Over half of the participants (59.4%) had an occupation related to the forest, 75.7% had a history of going to the forest, but a very small numbers (4.4%) had to spend the night at the forest. Roughly about 40% also admitted to use the bed net while sleeping (Table 2). Most of the participants (86.3%) had a house within a radius of 500 m from the forest, and the houses mostly constructed with wooden wall (35.7%), cement floor (56.1%) and corrugated iron roofs (82.2%). None of the previously reported risk factors [17–19] to malaria was associated with submicroscopic malaria infection.

**Table 1:** Demographic characteristic of participants

Demographic charateristic	n	%
Total	342	100
Gender		
Male	108	31.6
Female	234	68.4
Age group		
0–5	28	8.2
6–18	56	16.4
> 18	258	75.4
Study village		
Garunggang	67	19.6
Telagah	53	15.5
Bunga Rinte	51	14.9
Parit Bindu	73	21.3
Adin Tengah	25	7.3
Pajok	73	21.3
Ethnicity		
Batak Karo	334	97.7
Others (Jawa, Malay)	8	2.3
Occupation		
Forested occupation	213	59.4
Non-forested occupation	113	33.0
Housewives	26	7.6
Anemia (Hb < 11 g/dl)	28	8.2
Fever	2	0.5

**Table 2:** Risk factors to malaria infection

Risk factors	n	%
House construction		
Floor		
Cement	192	56.1
Soil	68	19.9
Other	82	24
Roof		
Corrugated iron	281	82.2
Thatch	39	11.4
Other	22	6.4
Wall		
Wooden	122	35.7
Bamboo	96	28.1
Brick	74	21.6
Other	50	14.6
House and forest within 500 m radius	295	86.3
Occupation related to the forest	203	59.4
History going to the forest	259	75.7
History of spend the night at the forest	15	4.4
Bed net ownership	144	42.1

### Prevalence of malaria infection

**Fig. 2:**
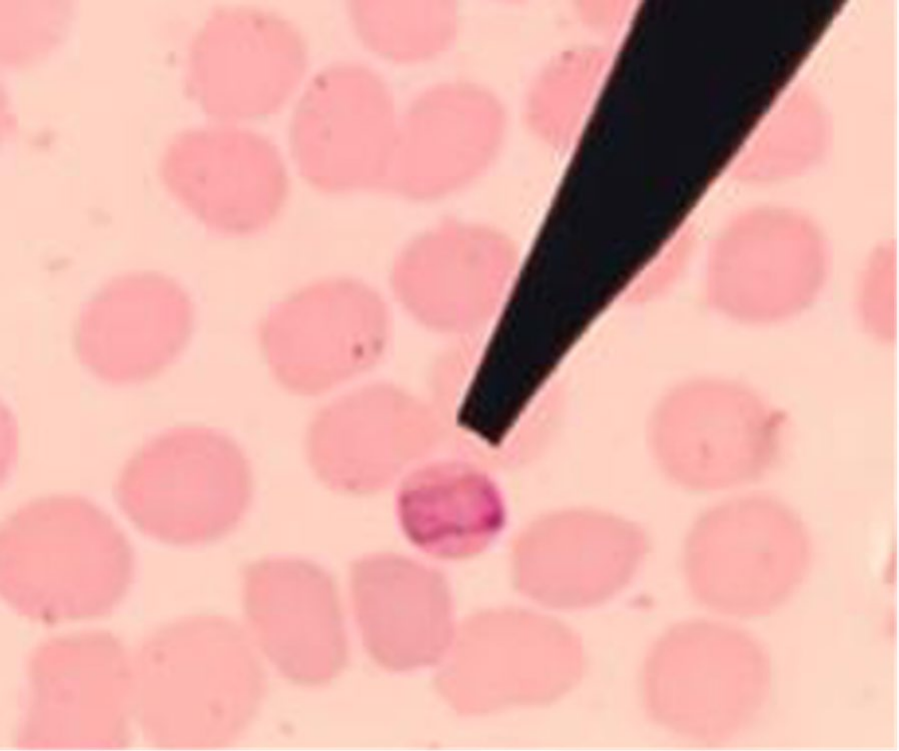
Microscopic examination shows band form of *Plasmodium malariae*

The prevalence of malaria by microscopy, RDT and PCR is shown in Table 3. Only one individual (0.3%) was positive for *P. malariae* by microscopy*,* with asexual and sexual parasite densities of 1160 and 2320 parasites µ/L of blood, respectively (Fig. 2). No positive result from RDT examination. Using molecular analysis, three additional submicroscopic infections were detected. Although the *P. malariae* detected by microscopy came up undetected molecularly (DNA concentration of 31.050 ng/μl). None of the common human malaria parasites were detected, while *P. knowlesi* and *P. malariae* infections contributing to 1.2% positivity. All of the infections occurred in women aged > 18 years with asymptomatic clinical manifestation and one respondent had an anaemia (Hb level was 8.7 g/dl).

**Table 3:** Prevalence of malaria infections among the population (N = 342) living in Langkat district, North Sumatera Province, Indonesia

Malaria diagnosis methods	Prevalence, n (%)	Species
Microscopy	1 (0.3)	*P. malariae*
RDT	0	0
PCR		
18sRNA	1 (0.3)	*P. malariae*
*sicavar*	2 (0.6)	*P. knowlesi*

## Discussion

This study was done as a follow-up study conducted at the same study sites in 2015. A significant decline was observed in the prevalence of microscopic malaria in the area from 16.5% to 0.3%. The dramatic reduction was also seen in the submicroscopic infection [11]. This suggested a successful control efforts in the near elimination settings in North Sumatera province, Indonesia. The absence of the most common human malaria infections, *P. falciparum* and *P. vivax*, indicating the control interventions for these species are effective.

The malaria control programme named “Gebrak Malaria” was started in 2009 with several control measures, such as active and passive case finding, mass blood surveys and malariometric surveys, effective drug administration, vector control and surveillance. These interventions were conducted to reduced malaria burden and accelerate the target of Indonesia being malaria-free by 2030 [20].

Both microscopic and submicroscopic examination in this study found non-falciparum malaria. Previously, a significant reduction of falciparum malaria infection in the population has been associated with an increased in the prevalence of *P. malariae* and *P. ovale* by 2 and 6 times, respectively [9]. *Plasmodium malariae* infection causes prolonged infection, but also reactivation after decades, and remain in the body as asymptomatic infection [21]. Further, its 72-h intra-erythrocytic cycle may cause this species to evade anti-malarial exposure [22]. Studies in Ghana, Uganda and Indonesia have reported *P. malariae* recurrences after 3-days treatment with ACT, suggesting that the universal treatment for malaria might not be appropriate enough to support human malaria elimination particularly for *P. malariae* [23–25].

The continuing trends of decreased incidence in human-only malaria in Sabah, and an increased in knowlesi malaria explains that the current control measures are mainly addressing human-malaria only. *P. knowlesi* has more complex factors contributing in its life cycle, involving humans, the mosquito vectors and the macaque hosts. Environmental factors such as deforestation and land-use change contributed to more competent vectors, increased breeding sites which in favour to an increase in knowlesi transmission [18, 26, 27]. Hence, the standard malaria prevention is not sufficient, and there is a need of *P. knowlesi* specific control measures.

There were several limitations in this study. First, all malaria infections detected occurred in females. The participants were screened for only one day in each survey area, and screening was done between 9 am and 4 pm. The study sites were located in the middle of the forest, and most of the male residents work in the forest and return to the village at dawn. This may have increased female participation (68%) and caused a sampling bias in this study. Although a low proportion of *P. knowlesi* infection was detected among females, knowlesi malaria is mostly found in men [28], who have more exposure to forestry and agricultural activities [17]. *Plasmodium knowlesi* infection is also more common in adults and usually among older individuals compared to falciparum and vivax malaria [28]. The presence of asymptomatic infections in these individuals could be due to the immunity to the malaria parasite [17].

A very low prevalence of malaria was found in this study. Surprisingly, the only parasite detected by microscopy resulted in a negative PCR. One of the possibilities was DNA degradation due to improper collection and storage of the filter papers [6, 8]. Filter paper stored at 30 °C with 60% humidity reduced the sensitivity by 100 times than the whole blood samples on PCR detection of malaria parasites [29]. The study sites are located in remote villages in Langkat district with a long journey to the preferred research laboratory. The storage of the samples were at room temperature before being transported for examination, and might have affected the quality of the samples. Furthermore, the utility of an RNA-preserving medium to store blood samples in areas distant to a laboratory, the use of more sensitive RNA extraction protocol and PCR assay using different gene targets and methods may increase the sensitivity of malaria detection, and may exclude a possible false-negative finding in this study [30, 31].

Despite the limitations of this study, the possibility of successful malaria control resulting in significant decline of malaria burden in these areas was could not completely ruled out. The incidence of microscopic and submicroscopic malaria in the same study areas in 2015 were 33.5% and 13.6%, respectively. Four *Plasmodium* species except *P. ovale* were detected [12]. In contrast, the malaria prevalence by microscopy and PCR were already at a very low level. Only one sample was positive by microscopy, and none of the common human malaria was detected. This supported the hypothesis that the malaria control measures have been successful in this study sites. Furthermore, malaria transmission is very sensitive to weather changes with the transmission peak at the end of the rainy season [32]. This study was conducted during the dry season, while the samples collection in the 2015 study was during the rainy season [12, 13], therefore further decrease in malaria prevalence may occur. The use of serological detection may explain the trend of malaria transmission in the study areas.

## Conclusion

Indonesia aims to become malaria free area by 2030. Follow-up survey in Langkat district, North Sumatera province showed a substantial decrease of malaria prevalence to 1.2%. Submicroscopic infection contributed to 75% of these malaria cases. *Plasmodium malariae* and *P. knowlesi* were the only malaria species detected suggesting the control efforts for human malaria may be effective in reducing the malaria burden in the area. Additional strategies are still needed to accelerate malaria elimination by intensification of vector control, targeting the submicroscopic malaria, and the interruption of the non-human malaria parasite transmission, thus Indonesia can achieve its target to be malaria free.

## Data Availability

The dataset used and/or analysed during the current study are available from the corresponding author upon request.

## Abbreviations

ACD: Active case detection
MBS: Mass blood survey
PCR: Polymerase chain reaction
RDT: Rapid diagnostic test
RBM: Roll Back Malaria
SDGs: Sustainable Development Goals
